# FSH Modulates PKAI and GPR3 Activities in Mouse Oocyte of COC in a Gap Junctional Communication (GJC)-Dependent Manner to Initiate Meiotic Resumption

**DOI:** 10.1371/journal.pone.0037835

**Published:** 2012-09-13

**Authors:** Junxia Li, Guankun Mao, Guoliang Xia

**Affiliations:** State Key Laboratory for Agro-Biotechnology, College of Biological Science, China Agricultural University, Beijing, China; Institute of Zoology, Chinese Academy of Sciences, China

## Abstract

Many studies have shown that cyclic adenosine-5′-monophosphate (cAMP)-dependent protein kinase A (PKA) and G-protein-coupled receptor 3 (GPR3) are crucial for controlling meiotic arrest in oocytes. However, it is unclear how gonadotropins modulate these factors to regulate oocyte maturation, especially by gap junctional communication (GJC). Using an *in vitro* meiosis-arrested mouse cumulus-oocyte complex (COC) culture model, we showed that there is a close relationship between follicle-stimulating hormone (FSH) and the PKA type I (PKAI) and GPR3. The effect of FSH on oocyte maturation was biphasic, initially inhibitory and then stimulatory. During FSH-induced maturation, rapid cAMP surges were observed in both cumulus cells and oocyte. Most GJC between cumulus cells and oocyte ceased immediately after FSH stimulation and recommenced after the cAMP surge. FSH-induced maturation was blocked by PKAI activator 8-AHA-cAMP. Levels of PKAI regulatory subunits and GPR3 decreased and increased, respectively, after FSH stimulation. In the presence of the GJC inhibitor carbenoxolone (CBX), FSH failed to induce the meiotic resumption and the changes in PKAI, GPR3 and cAMP surge in oocyte were no longer detected. Furthermore, GPR3 was upregulated by high cAMP levels, but not by PKAI activation. When applied after FSH stimulation, the specific phosphodiesterase 3A (PDE3A) inhibitor cilostamide immediately blocked meiotic induction, regardless of when it was administered. PKAI activation inhibited mitogen-activated protein kinase (MAPK) phosphorylation in the oocytes of COCs, which participated in the initiation of FSH-induced meiotic maturation *in vitro*. Just before FSH-induced meiotic maturation, cAMP, PKAI, and GPR3 returned to basal levels, and PDE3A activity and MAPK phosphorylation increased markedly. These experiments show that FSH induces a transient increase in cAMP levels and regulates GJC to control PKAI and GPR3 activities, thereby creating an inhibitory phase. After PDE3A and MAPK activities increase, meiosis resumes.

## Introduction

Physiologically, even if fully grown oocytes in follicles acquire complete maturation potential, they remain at the germinal vesicle (GV) stage before the gonadotropin surge. Similar to *in vivo* resumption of meiosis in response to gonadotropin signaling, oocytes undergo meiotic maturation spontaneously when liberated from the follicle into a suitable culture medium. Maintaining high intracellular levels of cyclic adenosine-5′-monophosphate (cAMP) by modulation activity of adenylyl cyclase (AC), which catalyzes the synthesis of cAMP, or phosphodiesterase (PDE), which hydrolyzes cAMP, or by supplementing the culture medium with cAMP analogs, will arrest oocytes in the prophase during the first meiotic division [1], [2], because it is believed that a consistently high concentration of cAMP in mammalian oocytes is an important factor for maintaining maturation arrest.

Elevated levels of cAMP in the oocyte maintain meiotic arrest by activating protein kinase A (PKA). PKA regulates proteins that control the activity of cyclin-dependent kinase 1 (CDK1) [3]. These proteins include the phosphatase, CDC25 [4], [5], and the kinase, WEE IB [6]. When CDK1 is phosphorylated, it is inactive, and oocytes become arrested in the prophase. However, when it is dephosphorylated by CDC25, it becomes active, and the prophase-to-metaphase transition occurs [5], [7]. Thus, high levels of cAMP in the oocyte keep PKA active and CDK1 inactive, while low levels of cAMP reduce PKA activity and allow CDK1 activation and meiotic progression. *In vivo*, cAMP in oocyte is supplied not only by follicle cells, through gap junctional communication (GJC), but also by oocyte itself. They are all necessary for meiotic arrest. The mouse oocyte generates cAMP through the G-protein-coupled receptor 3 (GPR3) [8], [9], [10], [11]; the Gs G-protein [12], and AC [13]. If any of these components is inhibited or removed from the oocyte, germinal vesicle breakdown (GVBD) proceeds spontaneously. Even if additional cAMP generated in somatic cells enters the oocyte, the amount is insufficient to maintain meiotic arrest in the absence of GPR3 in the oocyte [9], [10], [11], [14]. Recently, it was reported that mouse mural granulosa cells express natriuretic peptide precursor type C (NPPC) messenger RNA (mRNA), whereas cumulus cells express mRNA encoding the NPPC receptor NPR2, a guanylyl cyclase. NPPC increases cGMP levels in cumulus cells and oocytes, and inhibits the resumption of meiosis *in vitro* [15].

Meiotic maturation in cumulus-oocyte complexes (COCs), but not denuded oocytes (DOs), can be induced *in vitro* by follicle-stimulating hormone (FSH). The FSH-induced COC model is generally used to study mechanisms of gonadotropin-induced oocyte maturation, because LH receptors are absent or expressed at very low levels in cumulus cells, and LH has no effect on mouse COC maturation [16], [17].

A cAMP surge in the follicle or COC may trigger GVBD following stimulation with LH or FSH. It has been hypothesized that a certain concentration of cAMP is maintained in GV oocytes, while a transient increase in cAMP induced by hormonal stimulation is likely to trigger GVBD [18]. The dramatic change in cAMP levels may be an important stimulus for meiosis reinitiation but not the absolute cAMP levels in the oocyte. When gonadotropic hormones activate their receptors, cAMP is produced in cumulus cells. *In vitro* studies have shown that FSH induces an increase in cAMP levels in cumulus cells and in the oocyte via diffusion from somatic cells through GJC [19]. However, the meiotic resumption must involve a decrease in oocyte cAMP levels. The uncoupling of cumulus cells from oocytes by interruption of gap junctions accompanied by cumulus expansion may block the elevation of intra-oocyte cAMP. PDE3A, the major cAMP-hydrolyzing PDE present in the oocyte, may degrade cAMP in oocytes. As the hydrolytic capacity of PDE far exceeds the maximum rate of synthesis by AC, cellular levels of cAMP are thought to be more sensitive to inhibition of PDE than to changes in AC activity. Thus, an increase in PDE enzyme activity may be involved in the resumption of meiosis.

PDE3A has been shown to play a critical role in mouse oocyte maturation; microinjection of active PDE into isolated mouse oocytes arrested with the PDE inhibitor IBMX caused GVBD [20]. The oocytes of female *Pde3A* ^−/−^ mice are unable to undergo meiotic resumption despite normal follicular growth and ovulation. Likewise, oocytes from *Pde3A* ^−/−^ mice do not spontaneously mature when released from the follicles into culture medium. The ability of these oocytes to resume meiosis is restored by PKA inhibition or by microinjecting RNA encoding the phosphatase CDC25 [21] and inactivation of Gs or downregulation of the GPR3 can also restore meiotic resumption in the *Pde3A^−/−^* oocytes [14]. Moreover, 5′-AMP, the product of PDE activity, may serve as a transducer of the meiotic induction process by stimulating AMP-activated protein kinase [22].

Several studies have revealed that FSH has a biphasic effect on oocyte maturation; after adding FSH, oocyte maturation was initially inhibited and then stimulated [23], [24], [25]. However, the mechanism involved remains unclear. Although PKAI, GPR3, and PDE3A have been shown to participate in meiotic arrest in oocytes, their relationships with FSH are not well understood.

The aim of the present study was to determine the molecular mechanisms underlying the biphasic effect of FSH on oocyte maturation, especially the possible correlations among GJC, PKAI, GPR3, PDE3A and mitogen-activated protein kinase (MAPK), and to elucidate the signal transduction pathways involved in this process. Finally, to better understand the mechanisms of gonadotropin-induced meiotic resumption.

## Results

### FSH increases cAMP levels in both the oocyte and cumulus cells of the cumulus- oocyte complex (COC) and blocks oocyte-cumulus cell GJC

**Figure 1:**
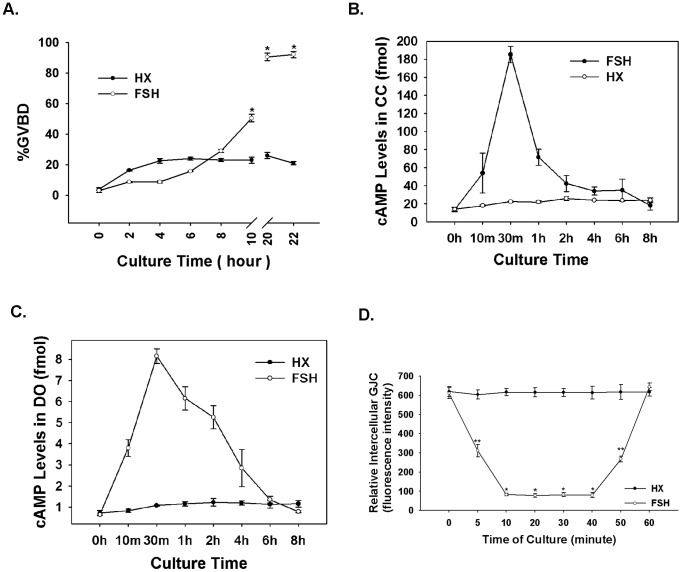
FSH increases cAMP levels and blocks oocyte-cumulus cell gap junctional communication (GJC). (A) The biphasic effect of FSH on oocyte maturation. Cumulus-oocyte complexes (COCs) were cultured with or without 50 IU/L FSH in HX medium for 22 h. The rate of GVBD was scored in 2-h intervals during culture. FSH inhibited maturation during the first 8 h of the treatment period and stimulated maturation after 8 h. *P<0.01 vs. the control group for that time point. (B) Changes in cAMP levels in cumulus cells of COCs after FSH treatment. (C) Changes in cAMP levels in the oocytes of COCs stimulated with FSH. (D) Cumulus cell-oocyte GJC during FSH-induced *in vitro* oocyte maturation. COCs were cultured in HX medium containing FSH (open circles) or not containing FSH (filled circles) for various periods of time. Then they were pulsed with calcein-AM, denuded of surrounding cumulus cells, and assessed for intra-oocyte fluorescence. A mean of 10 oocytes were used at each time point in each of three replicate experiments. **P<0.05, *P<0.01 vs. control.

As observed previously, hypothaxing (HX) maintained meiosis arrest in most COCs in the present study. FSH had a biphasic effect on oocyte maturation, i.e., inhibitory during the first 8 h and stimulatory after 8 h (Figure 1A).

To determine why FSH initially inhibited oocyte maturation, mouse COCs were cultured in control HX medium or HX medium supplemented with 50 IU/L FSH. At various time points, oocytes and cumulus cells were collected and analyzed for cAMP concentrations by radioimmunoassay (RIA). There was a notable cAMP surge not only in cumulus cells, but also in oocytes, approximately 30 min after FSH stimulation. However, the change in cAMP level was much greater in cumulus cells than in oocytes. In control COCs (not treated with FSH), cAMP levels increased only slightly (Figure 1B and 1C).

During FSH-induced COC maturation, GJC between the oocyte and the cumulus cells decreased sharply during the first 10 min of culture, thereafter remaining stable for 40 min, before increasing to initial levels at 60 min. Compared to COCs cultured in the presence of FSH, GJC in control COCs remained high at all time points (Figure 1D).

### The PKAI activator 8-AHA-cAMP inhibits FSH-induced meiotic resumption in COCs but only when applied within 4 h after FSH stimulation

**Figure 2:**
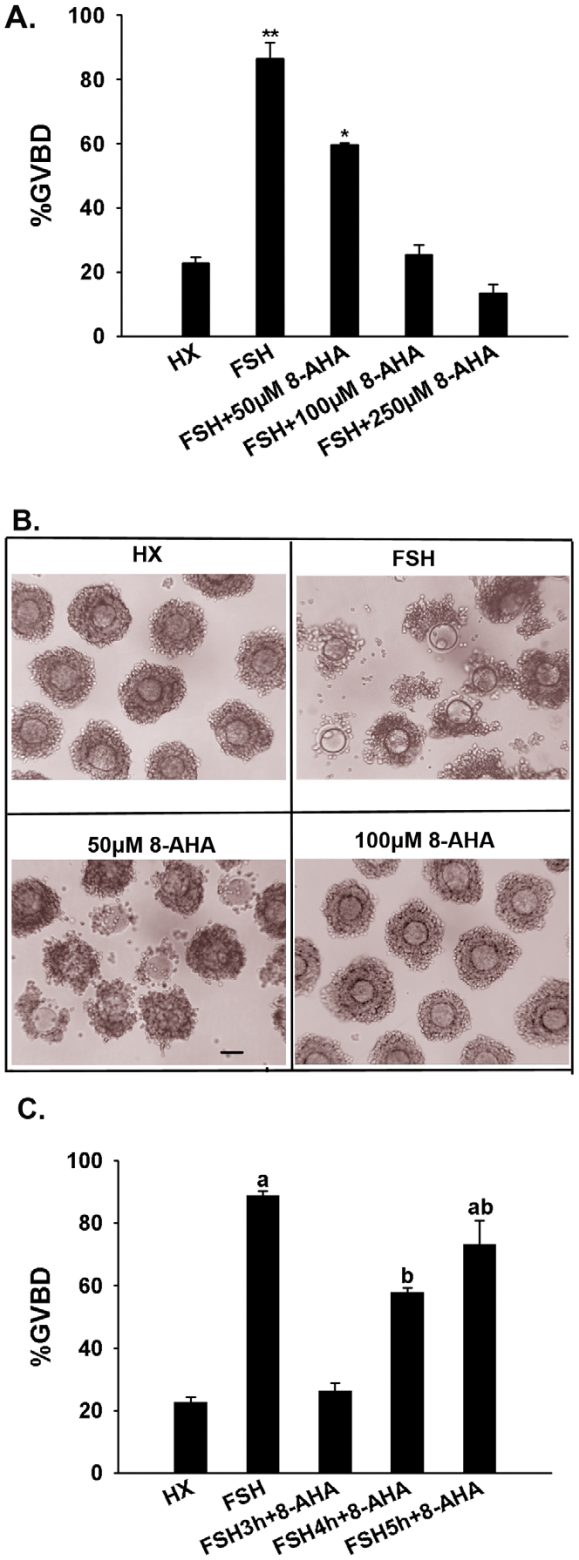
Effect of PKAI on the FSH-induced resumption of meiosis in COC. (A) The PKAI activator 8-AHA-cAMP (8-AHA) dose-dependently inhibited the FSH-induced resumption of meiosis in COC. (B) Cumulus expansion. Cumulus expansion was detected at the end of 22 h culture. HX, control; FSH, 50 IU/L FSH; 50 µM 8-AHA-cAMP; 100 µM 8-AHA-cAMP. *Scale bar*, approximately 50 µm. (C) The crucial period for the effect of PKAI on FSH stimulation. When 8-AHA-cAMP was added to cultures 3 or 4 h after the start of culture, meiotic induction was significantly reduced. Administration of 8-AHA-cAMP 5 h after the start of culture did not inhibit the induction of meiosis. Columns with different superscripts are significantly different (*P*<0.05).

The specific PKAI activator 8-AHA-cAMP inhibited FSH-stimulated meiotic resumption and cumulus expansion in a dose-dependent manner in COCs. The maturation rate of COCs treated with 100 µM 8-AHA-cAMP was similar to that of COCs treated with HX alone (Figure 2A).

To define the time period during which PKAI activity modulated the effects of FSH stimulation, COCs were cultured in the presence of 4 mM HX and 50 IU/L FSH. In one set of cultures, 250 µM 8-AHA-cAMP was added 3, 4, 5 h after the start of culture. The oocytes in all of these groups were examined after 22 h of culture for GVBD. When 8-AHA-cAMP was added to cultures before 4 h from the start of culture, meiotic induction was significantly reduced. In contrast, administration of 8-AHA-cAMP after 4 h did not inhibit meiotic induction (Figure 2B).

### FSH regulates levels of the regulatory subunit of PKAI (RI) during its inhibitory phase, whereas activation of PKAI results in down-regulation of its RI

**Figure 3:**
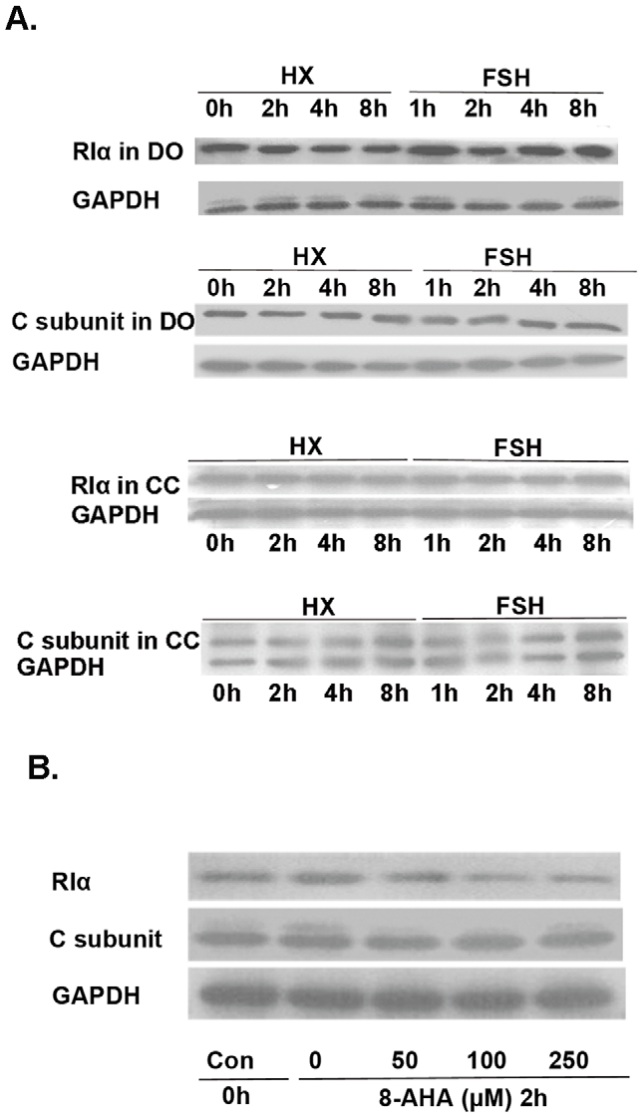
Effects of FSH on PKAI, and decrease in PKAI regulatory subunit (RIα) levels after activation of PKAI. (A) FSH reduced PKAI regulatory subunit RIα levels, but not levels of the catalytic subunit (C) of PKAI, in oocytes of COCs (DOs), but not cumulus cells (CCs), 2 h after the start of culture. DOs and CCs from 200 COCs treated/not treated with FSH were collected at various time points and analyzed by Western blotting. (B) PKAI activation resulted in a decrease in RIα levels in oocytes. COCs were incubated in HX medium supplemented with increasing concentrations of 8-AHA-cAMP (0–250 µM) for 2 h. Oocytes of COCs were collected at 2 h for Western blotting analysis of PKAI RIα and C levels. 8-AHA-cAMP reduced oocyte RI levels in a dose-dependent fashion, but had no obvious effect on PKAI C levels. Western blotting experiments were performed at least three times with similar results. The blot shown is representative of three experiments.

Western blotting analysis showed that, under conditions of FSH stimulation, RI levels in DOs, but not cumulus cells, were significantly reduced at 2 h and subsequently increased. In control cultures, RI levels decreased gradually during 0–8 h of culture (Figure 3A and Figure S1A). PKA catalytic subunit (C) levels showed no obvious changes.

We next attempted to define the relationship between PKAI activation and RI downregulation. The 8-AHA-cAMP treatment reduced RI levels in oocytes in a dose-dependent fashion, but had no obvious effect on PKA C subunit levels (Figure 3B and Figure S1B).

### GPR3 expression is increased by FSH at 2 h and is induced by activation of AC but not PKAI

**Figure 4:**
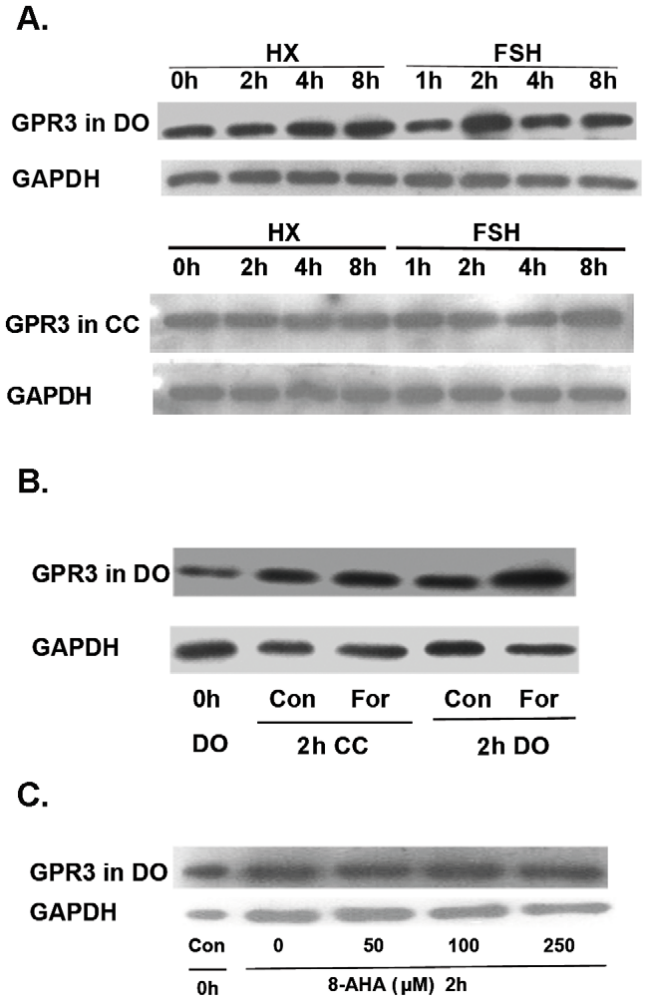
GPR3 levels are increased by FSH, as well as the activation of AC but not PKAI. (A) FSH increased GPR3 levels in DOs, but not CCs, at 2 h, during the resumption of meiosis. DOs and CCs from 200 COCs treated/not treated with FSH were collected at various time points and analyzed by Western blotting. (B) Forskolin (For) upregulated GPR3 levels in DOs, but not CCs. COCs were incubated in HX medium supplemented with 5 µM forskolin for 3 h. Oocytes of COCs were collected at 2 h for Western blot analysis of GPR3 levels. (C) Direct activation of PKAI had no effect on the change in GPR3 levels. COCs were incubated in HX medium supplemented with increasing concentrations of 8-AHA-cAMP (0–250 µM) for 2 h; Oocytes of COCs were collected at 2 h for Western blotting analysis of GPR3. Western blotting experiments were performed at least three times with similar results. The blot shown is representative of three experiments.

To evaluate the relationship between FSH and GPR3 before the initiation of meiosis, we used the same FSH-induced maturation model and Western blotting analysis. The effect of FSH on GPR3 expression was opposite to its effect on PKA RI levels. Levels of GPR3 were markedly increased in DOs of COCs treated with FSH for 2 h. No change was detected in cumulus cells (Figure 4A and Figure S2A). To determine which factors may control GPR3 expression, we treated COCs with the AC activator forskolin. We found that AC activation contributed to the upregulation of GPR3 in oocytes, but not in cumulus cells (Figure 4B and Figure. S2B). However, there was no notable change in GPR3 levels after PKAI was directly activated (Figure 4C and Figure S2C).

### The Effect of FSH on PKAI, GPR3 and cAMP surge in oocyte needs GJC

**Figure 5:**
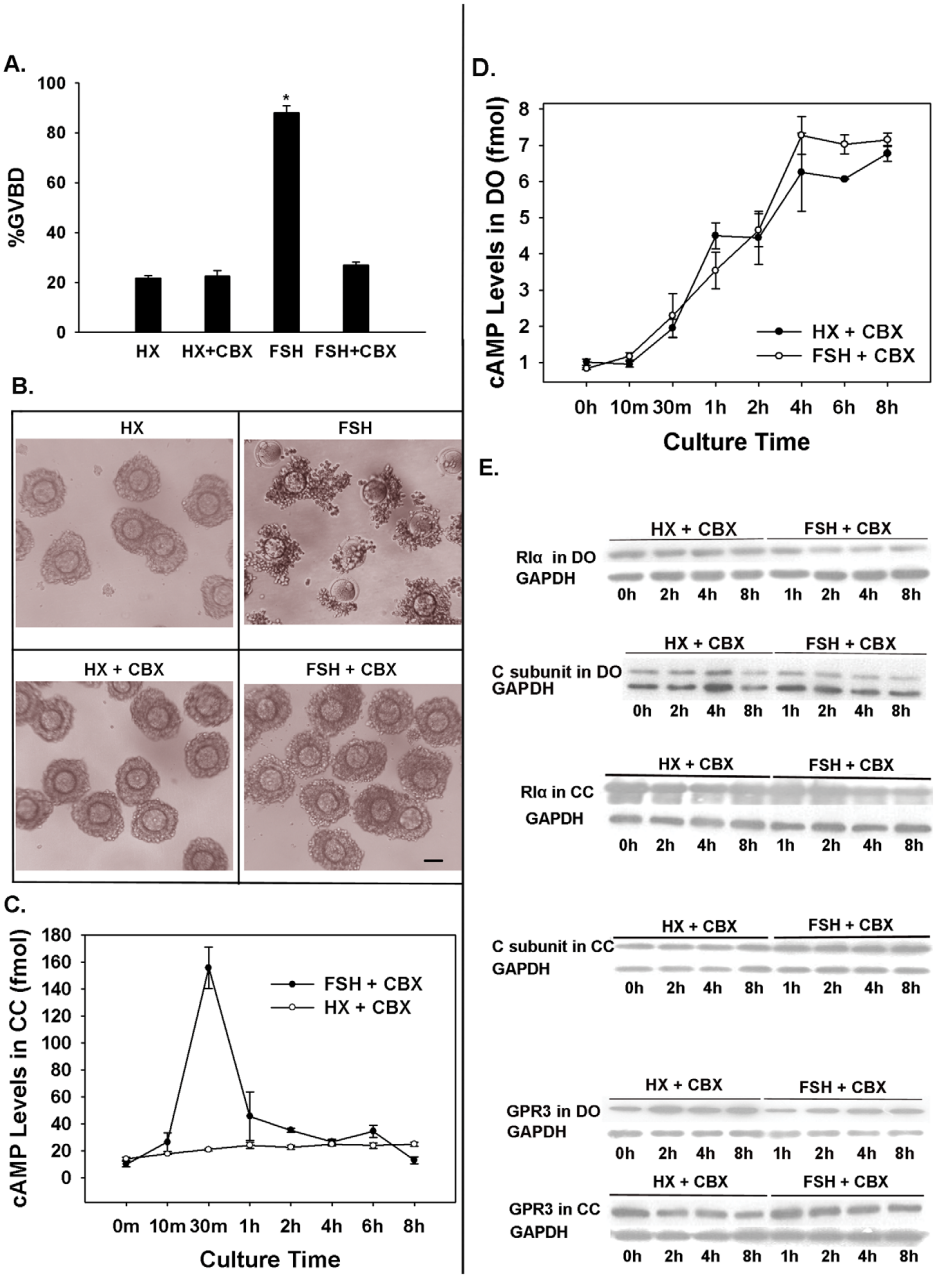
The effects of GJC on PKAI, GPR3 levels and cAMP surge in oocytes of COCs stimulated by FSH. (A) Carbenoxolone (CBX) completely suppressed FSH-induced GVBD. COCs were preincubated for 0.5 h in HX medium containing 100 µM CBX. Then FSH was added to some COC cultures, and the cultures were maintained for 22 h. Percent GVBD was then determined. The bar marked with the asterisk is significantly different from the other bars. (B) CBX suppressed FSH-induced cumulus expansion of COC. HX, control; FSH, 50 IU/L FSH; HX+CBX, HX medium supplemented with 100 µM CBX; FSH+CBX, HX medium supplemented with 50 IU/L FSH and 100 µM CBX. *Scale bar*, approximately 50 µm. (C) cAMP levels in cumulus cells (CCs) of COCs preincubated with CBX and then treated with FSH. (D) cAMP levels in oocytes of COCs preincubated with CBX and then treated with FSH. A mean of 100 COCs were used at each time point in each of three replicate experiments. The mean ± SEM of three individual experiments are shown. (E) Inhibition of oocyte-cumulus cell GJC abolished FSH-induced changes in PKAI and GPR3 levels. COCs were preincubated for 0.5 h in HX medium containing 100 µM CBX. Then FSH was added to the culture medium. DOs and CCs from 200 COCs treated/not treated with FSH were collected at various time points and analyzed by Western blotting. The blot shown is representative of three experiments.

In this experiment, BSA was replaced with polyvinylpyrrolidone (PVP) because carbenoxolone (CBX) is less effective in BSA-supplemented medium. COCs were pretreated for 30 min in HX medium containing 100 µM CBX to block GJC. FSH was then added to cultures, which were maintained for 22 h to examine percentage maturation, or harvested at various time points to detect cAMP concentrations or changes in PKAI or GPR3 levels by Western blotting. In the CBX treatment groups, about 26.90% of the COCs underwent maturation, compared to 88.03% of COCs not treated with CBX (Figure 5A). CBX completely suppressed FSH-induced GVBD and cumulus expansion (Figure 5B).

As expected, the changes in PKAI and GPR3 levels were abolished by the blockade of oocyte-cumulus cell GJC, and no cAMP surge could be detected in the oocytes of FSH-stimulated COCs. Changes in GPR3 and RI protein levels were similar to those in the control groups in experiments 3 and 4 (Figure 5E and Figure S3). There was still a notable cAMP surge in cumulus cells about 30 min after FSH stimulation. In oocytes, cAMP levels increased gradually, which was similar to that in the control group (not treated with FSH) (Figure 5C and 5D).

### PDE3A inhibition blocks the effect of FSH on mouse oocyte maturation, but not cumulus expansion

**Figure 6:**
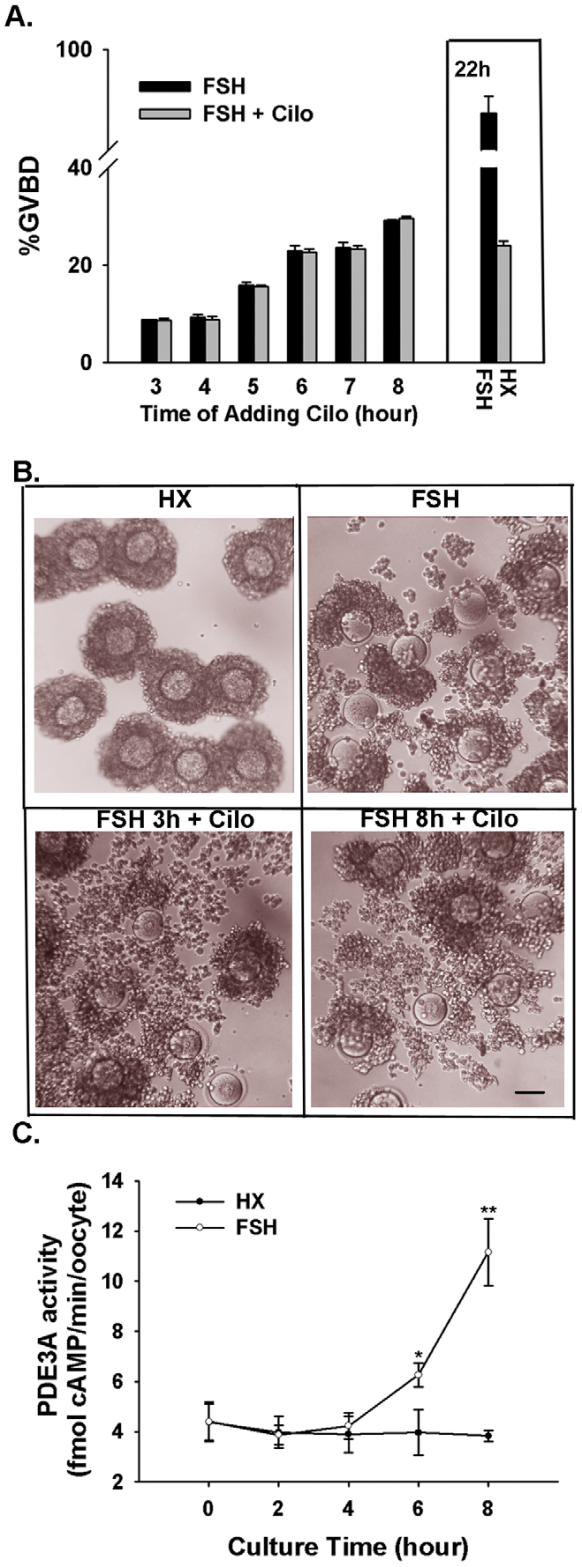
Correlations between FSH and PDE3A. (A) Inhibiting PDE3A blocked the effect of FSH on maturation. COCs were cultured in HX medium containing 50 IU/L FSH. Cilostamide (cilo) was added to one set of cultures 3, 4, 5, 6, 7, or 8 h after the start of culture. GVBD was assessed at 22 h (gray bars). The other set of cultures was examined at different intervals up to 8 h (black bars). Cilostamide blocked further meiotic maturation at each time point at which it was administered. GVBD percentage of the controls is indicated by the box. (B) Inhibiting PDE3A didn't affect cumulus expansion induced by FSH. Cumulus expansion was detected at the end of 22 h culture. HX, control; FSH, 50 IU/L FSH; FSH 3 h+Cilo, Cilostamide (cilo) was added to culture 3 h after the start of FSH treatment. FSH 8 h+Cilo, Cilostamide (cilo) was added to culture 8 h after the start of FSH treatment. *Scale bar*, approximately 50 µm. (C) After stimulation with FSH for 6 h, oocyte PDE3A activity significantly increased. Means marked with asterisks are significantly different (*P<0.05, **P<0.01).

COCs were cultured in 4 mM HX and 50 IU/L FSH. In one set of cultures, 2 µM cilostamide was added at different time points after the start of culture. The oocytes in all of the groups were examined at 22 h for GVBD. As shown in Figure 6A, when cilostamide was added to the cultures, meiotic induction was immediately blocked, regardless of the time point at which cilostamide was administered, i.e., the percentage of GVBD at 22 h was identical to that at the time of cilostamide administration (Figure 6A). However, cumulus expansion was normal (Figure 6B).

**Figure 7:**
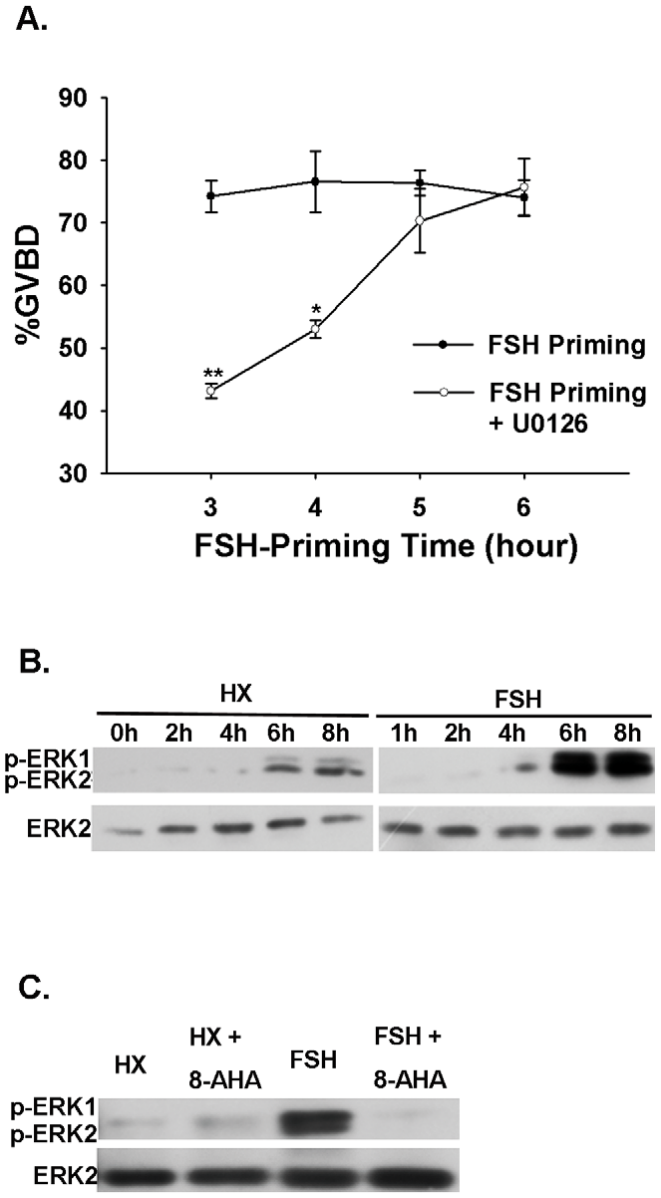
Correlations between FSH and MAPK and PKAI in oocytes. (A) Activation of MAPK in the oocytes of COCs was required for FSH-induced meiotic maturation. DOs from the COCs primed with FSH for 3, 4, 5, or 6 h were transferred to fresh HX medium containing the specific MAPK inhibitor U0126 and were further cultured for a total 22 h. U0126 significantly inhibited GVBD in DOs. Means marked with asterisks are significantly different (*P<0.05, **P<0.01). (B) FSH-activated MAPK in oocytes of COCs. Oocytes of COCs were collected after treatment with FSH for 0, 2, 4, 6, or 8 h for Western blot analysis of MAPK phosphorylation. (C) PKAI activation inhibited FSH-induced MAPK phosphorylation in oocytes of COCs. Oocytes from COCs were collected 8 h after treatment for Western blot analysis of MAPK phosphorylation. DOs from 100 COCs were collected at various time points for Western blotting. The blot shown is representative of three experiments.

MAPK pathway in oocyte participates in the regulation of FSH-induced COC meiotic resumption We pretreated COCs with FSH for different periods of time and then stripped the cumulus cells away. Then we cultured the denuded oocytes in fresh HX medium without FSH for 22 h. The percentage of maturation of DOs from COCs primed with FSH for 3 h or more was significantly higher (72.4% of COCs primed for 3 h) than in controls (26.67%) (Figure 7A, open circles).

DOs from COCs primed with FSH for 3, 4, 5 or 6 h were transferred to fresh HX medium containing the specific MAPK inhibitor U0126, and were further cultured for a total 22 h. U0126 significantly inhibited GVBD in the DOs. However, when U0126 was added after 5 h, the difference between the U0126 and control groups was non-significant (Figure 7A).

### PKAI activation inhibits MAPK phosphorylation in the oocyte of the COC

DOs from COCs were collected at 0, 2, 4, 6, 8 h for Western blotting analysis with anti-p-ERK1/2 and anti-ERK2 antibodies after culture in HX medium, HX medium containing FSH, or HX medium containing FSH and 8-AHA-cAMP. Only very low levels of MAPK phosphorylation were detected during culture in non-supplemented HX medium. FSH activated MAPK in oocytes of COCs, with phosphorylation reaching its maximum level at 8 h (Figure 7B). 8-AHA-cAMP significantly inhibited MAPK phosphorylation (Figure 7C).

### PDE3A activity increases significantly before the initiation of meiosis

To determine how PDE3A activity changes during FSH-induced oocyte maturation, we cultured COCs in HX medium or HX medium containing 50 IU/L FSH. At different time points, oocytes of COCs were collected and analyzed for PDE3A enzymatic activity. There was a significant increase in PDE3A activity in oocytes approximately 6 h after FSH stimulation. No obvious change in PDE3 activity was detected in control oocytes at any time point (Figure 6C).

## Discussion

FSH had a biphasic effect on oocyte maturation, initially inhibitory and then stimulatory. A large body of research has addressed the mechanism of stimulation induced by FSH. The aim of the present study was to investigate regulatory mechanisms during the inhibitory phase. We found that FSH controls oocyte maturation through the GJC-dependent modulation of the activities of PKAI and GPR3 in mouse COCs, and that the activities of MAPK and PDE3A are arrested until just before the resumption of meiosis.

In vivo, FSH induces follicular development, but not oocyte maturation. Studies from our lab have revealed that FSH increases the levels of NPPC in mural granulose cells and that of its receptor Npr2 in cumulus cells in vivo, as a result maintaining high levels of cAMP in oocyte itself and meiotic arrest [15]. However, in vitro, FSH also shows an inhibitory effect to meiotic resumption of COC during its early culture, although at the end of culture, FSH induces meiotic maturation. The one of the reasons may be that there are not mural granulosa cells for FSH-induced COC model, but NPPC on cumulus cells still exist. Although FSH can up regulate the levels of Npr2 in cumulus cells [26], NPPC decreased gradually after COCs are isolated from follicle. So, it may be one of the reasons that FSH has a biphasic effect on oocyte maturation in vitro.

The PKA holoenzyme is a tetramer consisting of two regulatory (R) subunits and two inactive catalytic (C) subunits. There are two major isoforms of PKA, types I and II, which are defined by the presence of an RI or RII subunit. When cAMP binds to the R subunits, active C monomers are released, which phosphorylate substrates. In mice, four R subunits (RIα, RIβ, RIIα, and RIIβ) and two C subunits (Cα and Cβ) originating from distinct genes are present. In mouse oocytes, only RI protein was detected by photoaffinity labeling, but RI and RII were detected in cumulus cells [27]. The predominant R subunit mRNA expressed in oocytes was found to be RIα mRNA; no RIβ or RIIβ mRNA was detected [28]. Downs and Hunziker-Dunn [27] demonstrated that elevation of type I PKA in the oocyte is related to the maintenance of meiotic arrest, while type II PKA mediates cAMP-stimulated cumulus expansion and meiotic resumption. Persistent activation of PKAI leads to suppression of spontaneous meiotic maturation. Our results show that activation of PKAI leads to suppression of FSH-induced meiotic maturation. This suppressive effect persisted until 4 h after initial exposure to FSH. RI levels in oocytes from COCs were significantly reduced at 2 h after stimulation with FSH, but subsequently increased. There was no obvious change in catalytic subunit levels. In cumulus cells, there was no change in RI levels.

In our study, the decrease in PKAI R levels in oocytes suggests the activation of PKAI, but the similar results have not been seen in other reports and it is easy understood that reducing the regulatory subunit levels would increase the population of free catalytic subunits that can phosphorylate substrates. While RIα levels in mouse oocytes were reduced to undetectable levels by RNA-interference, the oocytes resumed maturation because they also lacked Cα [28]. Therefore, modulation of PKA by knockdown technology and physiological methods may be different. Following FSH stimulation, although R levels did not change before 2 h, a >8-fold increase in cAMP over basal levels was observed at 30 min in oocytes. PKAI was activated as a result of this increase in cAMP levels. PKAI activation may have peaked at 2 h, because activation of PKAI after 4 h did not suppress maturation. Similar to our results, in previous studies on rat PC12 pheochromocytoma cells, forskolin induced 70% of total cellular PKA activity within 30 min and this effect persisted throughout 2 h of stimulation [29], [30]. Increased intracellular cAMP concentrations have been demonstrated to affect the stability of certain mRNA species, including PKA regulatory subunit RIα mRNA [31]. The decrease in RIα protein levels at 2 h may reflect the instability of RIα mRNA. While PKAI is more sensitive to slight increases in cAMP levels, PKAII responds preferentially to higher cAMP levels because RI has a 2- to 8-fold higher affinity for cAMP than RII [32]. We detected a >90-fold increase in cAMP over basal levels in cumulus cells after FSH stimulation, but only an 8-fold increase in cAMP levels in oocytes. Together with the presence of PKAII in cumulus cells, this may contribute to the different modulation of PKAI in oocytes and cumulus cells.

The effect of FSH on GPR3 expression in oocytes was opposite to its effect on PKA RI levels. Like PKAI, no change was detected in cumulus cells. GPR3 activates Gs, which stimulates AC in the oocyte to elevate cAMP levels. Thus, the elevated cAMP levels in oocytes of COCs after FSH stimulation may partly contribute to the change in GPR3 expression. Because forskolin can also upregulate GPR3, upregulation of GPR3 after FSH stimulation results from high cAMP levels. According to the generally accepted paradigm for cAMP-induced transcription of genes, cAMP activates PKA, which in turn phosphorylates cAMP responsive element (CRE)-binding protein (CREB), which mediates transcription of cAMP-responsive genes. We found two CREB-binding sites in the GPR3 gene promoter, one located 106 base pairs (bp) upstream of the transcription initiation site and the other 67 bp downstream of the transcription initiation site (http://www.sabiosciences.com/chipqpcrsearch.php?species_id=1&factor=CREB&gene=GPR3&nfactor=n&ninfo=n&ngene=n&B2=Search). Because most known functional CREs are located within 170 bp upstream of the transcription start site [33], the putative CRE located upstream of the GPR3 transcription initiation may be functional and cAMP-inducible. While we still do not know the role of the CRE located downstream of the transcription start site, we proved that activation of PKAI did not affect the expression of GPR3. In NG108-15 cells, forskolin-induced nuclear translocation of the catalytic subunits is only blocked by a type II PKA inhibitor, forskolin-triggered phosphorylation of CREB depends only on PKAII, and PKAI regulates a downstream cytoplasmic pathway leading to activation of other transcription cofactors that interact with phosphorylated CREB to induce gene transcription [34]. This means that both PKA isoforms are involved in the mediation of CREB-driven transcription in response to cAMP. Although levels of RIα mRNA are 10- to 40-fold higher than those of RIIα mRNA in oocytes [28], RII protein was detected in oocytes by immunofluorescence staining, and showed a punctuate distribution within the cytoplasm [35]. Although the amount of PKAII is very low, it may have a crucial role in oocytes (e.g., in the control of gene transcription). Based on our results, we propose that GPR3 is regulated by a positive feedback mechanism, i.e., when cAMP levels are elevated, GPR3 expression is upregulated and more cAMP is produced.

FSH receptors are only present in cumulus cells which are coupled to oocyte via GJC. GJC is necessary for normal oocyte growth [36] and, in particular, control of the meiotic cell cycle [37], [38], [39], [40]. Similar to a previous report on isolated rat follicles exposed to LH [41], our results show that GJC between oocytes and the cumulus cells was significantly reduced soon after FSH stimulation, and was gradually restored after the cAMP surge. Why is there also a cAMP surge in oocyte at 30 min after the stimulation of FSH? Our results show that during the time from 10 min to 40 min, although it is the lowest levels of GJC opening, there is still about 15% opening GJC compared with the initial levels, thus when the cAMP levels in cumulus cell change sharply, the cAMP levels in oocyte will be affected by that in cumulus, but due to a small amount of opening GJC, the change level of cAMP of oocyte is much less than that in cumulus cells.

Oocyte can communicate with cumulus cells through not only the cell-cell coupling pathway, but also paracrine pathways. Our results show that changes in cAMP, PKAI, and GPR3 levels in the oocyte followed the effects of FSH on cumulus cells. To distinguish the cell-cell coupling and paracrine pathways, we blocked oocyte-cumulus cell GJC and then treated oocytes with FSH. We found that the FSH-induced cAMP surge in the oocyte mainly depended on patent gap junctions with neighboring cumulus cells, and that inhibition of oocyte-cumulus cell GJC abolished the changes in PKA I and GPR3. This indicates that all of these responses require GJC. Although GPR3 mainly exists in oocytes, there seemed to be no cAMP-activated cumulus cell-derived GPR3 ligands with paracrine effects on oocytes in our study. The results presented above suggest that modulation of the closing and opening of GJC is important for gonadotropin-induced maturation, and that closing GJC continually results in FSH losing its power of inducing meiotic maturation, which may result from continual PKAI activation.

As mentioned above, PKA may inactivate Cdc25B during meiotic arrest. Inactive Cdc25B fails to activate MPF. PKA can also activate Wee1B, thereby leading to MPF inactivation. PKA/Cdc25B and PKA/Wee1B pathways most likely work in a synergistic manner to maintain an inactive Cdc2/cyclin B complex and regulate the resumption of meiosis in mouse oocytes [42]. However, our results show that activation of mitogen-activated protein kinase (MAPK) in oocytes is important for the oocyte maturationinduced by FSH. and that PKA could inhibit the activation of MAPK.

MAPK is more abundantly expressed in both oocytes and cumulus cells in later stages of meiosis. It is well known that gonadotropic stimulation of meiotic resumption depends on MAPK activation in the somatic compartment of the follicle, but not in the oocyte, while intra-oocyte MAPK cascade activation is more closely related to post-GVBD events such as meiotic spindle organization [43], [44]. Strong evidence comes from knockout studies. Oocytes of c-mos-knockout mice undergo spontaneous GVBD with an abnormal polar body [45], [46], but granulosa cell-specific knockout oocytes do not mature [47]. Treating COCs with MAPK inhibitors inhibits MAPK signaling not only in cumulus cells, but also in oocytes. It is thus difficult to determine which compartment is more important. Although the microinjection of oocytes can interfere with MAPK function, the technological barrier is relatively high. Our gonadotropin-priming model allowed us to confirm the importance of MAPK in the oocyte itself for the resumption of meiosis. In our study, U0126 was used to inhibit MAPK pathway, it inhibits the second kinase from the bottom of MAPK pathway, namely, MEK, but not the last, MOS. Now, there is not MEK^−/−^ oocyte, and none knows the effect of MEK on oocyte meiotic maturation. Thus, it is the possible reason that our result is different from that of mos knockout. The other possibility is that there is some compensatory mechanism in mos^−/−^ oocyte for meiosis resumption, which compensates the lost of MOS partly. In addition, we make sure that we can pick up the oocyte whose cumulus cells are excluded absolutely. Although we are not sure whether some projections of cumulus cell were still stay in zona after removing of cumulus cells, no evidence has shown that MAPK can pass through the projections. Thus, it really needs a deep study. Intra-oocyte MAPK was not activated by FSH after activation of PKAI, which suggests that FSH activation of type I PKA in oocytes is required for inhibition of not only MPF, but also MAPK. Previous work on NG108-15 cells demonstrated that activation of PKA by ethanol inhibits B-Raf kinase activity, resulting in decreased MAPK phosphorylation, i.e., inhibition of MAPK signaling [48]. We think that naturally, MAPK pathway in oocyte itself participates in the regulation of FSH-induced COC maturation and makes the process of GVBD more normal and successful than that of inhibition of MAPK.

Before the initiation of meiotic resumption, the activities of MPF and MAPK must be restored. This depends on inactivation of PKA, which in turn depends on a decrease in the cAMP level. PDE3A is the major cAMP-hydrolyzing PDE present in oocytes, and it is important for the maintenance of meiotic arrest and oocyte maturation. Therefore, it is not surprising that the PDE3A inhibitor cilostamide prevented FSH-induced maturation, regardless of the time at which it was administered. PDE3A clearly has a permissive role in the control of FSH-induced oocyte maturation, although the COCs still appeared fully expanded, which indicates that cumulus expansion can be independent of GVBD. During FSH-induced maturation, PDE3A activity is tightly controlled, increasing markedly immediately before the resumption of meiosis. However, the precise mechanism of PDE3A activation in oocytes is unknown.

In conclusion, the results of this study demonstrate that FSH, acting on cumulus cells of COCs, regulates the activities of PKAI and GPR3 in oocytes in a GJC-dependent manner. Active PKAI inhibits the activation of MAPK in oocytes, which is important for the FSH-induced meiosis resumption. Until immediately before the resumption of meiosis, PDE3A activity is not elevated, resulting in very low cAMP levels. Inactivation of PKAI results in the activation of MAPK and MPF, resulting in the meiotic resumption. These are the potential causes of the biphasic function of FSH. Our work links the inhibitory and stimulatory phases of FSH action and improves our understanding of the mechanisms of gonadotropin-induced meiotic resumption.

## Materials and Methods

### Experimental animals

Immature 21 to 23 days old Kunming White female mice (outbreed strain) were used for all experiments. All animal treatment procedures were approved by the Animal Care Committee of China Agricultural University (CAU). Mice were provided with water and chow *ad libitum* and housed in air–conditioned rooms illuminated for 12 h per day. Follicle development was primed by intraperitoneal injection of each mouse with 5 IU pregnant mare serum gonadotropin (PMSG) and mice were killed by cervical dislocation 46–48 h later.

### Chemicals

All reagents and chemicals used in this study were obtained from Sigma-Aldrich Corp (St. Louis, MO), unless otherwise indicated. FSH was prepared as stock solutions in filtered phosphate buffered saline (PBS) containing 0.1% BSA. Carbenoxolone (3b-hydroxy-11-oxoolean-12-en-30-oic acid 3-hemisuccinate, CBX) was prepared as stock solutions in PBS. U0126, 8-AHA-cAMP (catalog number: A2104 [27]), and Forskolin were prepared in dimethylsulfoxide (DMSO) and the final concentration of DMSO was less than 0.1%, which had no significant effect on oocyte maturation (data not shown). Polyclonal rabbit anti-PKA RI antibody (catalog number: sc-28893 [49]), polyclonal rabbit anti-PKA Cα antibody (catalog number: sc-903 [25]), Polyclonal rabbit anti-GPR3 antibody (catalog number: sc-68876 [50]), monoclonal mouse anti-p-ERK1/2, polyclonal rabbit anti-ERK2 antibody, monoclonal mouse glyceraldehyde-3-phosphate dehydrogenase (GAPDH) antibody, and horseradish peroxidase-conjugated second antibodies were purchased from Santa Cruz Biotechnology, Inc. (Santa Cruz, CA, USA).

### Oocyte isolation and culture

Ovaries were removed, placed in culture medium, and large antral follicles (300–400 µm) were punctured under a stereomicroscope with sterile 27-gauge needles. Cumulus-oocyte complexes (COCs) of equal size with several layers of cumulus cells were collected. In each experiment, one group COCs were cultured in a 100 µl drop covered with paraffin oil in a 35-mm culture dish.

Oocytes were cultured at 37°C, in an atmosphere of 5% CO_2_ and 100% humidity. After culture, oocytes were denuded mechanically and assessed for maturation scoring for GV (meiotic arrest), GVBD (meiotic resumption). The percent of GVBD (including polar body 1) per total number of oocytes (% GVBD) were calculated. Oocytes that had degenerated were excluded.

The culture medium used for this study was M199 (GIBCO-Invitrogen, Carlsbad, CA, USA) medium containing 4 mM HX; 0.23 mM sodium pyruvate; 2 mM glutamine; 3 mg/ml lyophilized crystallized BSA; 75 mg/ml potassium penicillin G; and 50 mg/ml streptomycin sulfate. This medium is designated as HX medium.

### Western blot analysis

Proteins from cumulus cells (CC) or denuded oocytes (DO) of COCs per sample were extracted with double-strength electrophoresis sample buffer after culture, supplemented with 1 mM phenylmethylsulfonylfluoride and 1 mM sodium orthovanadate for 20 min on ice, and stored at −80°C. Before electrophoresis, the lysates were heated to 100°C for 5 min, cooled down on ice immediately, and then centrifuged at 12,000 *g* for 5 min. The proteins were separated by SDS-PAGE with a 4% stacking gel and a 10% separating gel for 50 min at 160 V, and electrically transferred to a nitrocellulose membrane (Amersham Pharmacia Biotech, Braunschweig, Germany). The membrane was saturated with 5% nonfat dry milk and then incubated with corresponding antibody. Proteins were detected using SuperSignal West Pico™ (enhanced chemiluminescence) detection system (Pierce Chemical Co., Rockford, IL).

### Determination of cAMP by radioimmunoassay (RIA)

After different incubation time, COCs were denuded by manual pipetting with a small fine-bore pipette in HX medium containing 0.2 mM 3-isobutyl-1-methylxanthine (IBMX). CC and homogeneous DO were collected separately. In a volume less than 5 µ1, DOs isolated from the cultured COCs were transferred to 100 µ1 of 0.1 M HCl. CCs were centrifuged at 8,000 g for 5 min, and the supernatant (culture medium) were pipetted out and 100 µ1 of 0.1 M HCl was added. All samples were kept on ice for at least 10 min, and then stored at −80°C. An equal volume of medium was collected and used as a blank. Prior to RIA, samples were thawed and centrifuged at 12,000 g for 5 min and the supernatant was collected to a glass tube and dried in a 60°C oven. The entire samples were assayed for cAMP as the RIA kit (A Beckman coulter company, Immunotech, IM1117, USA) procedure described. Measured cAMP was proportional to the number of DOs or CCs, which is equal to the number of COCs.

### Oocyte-cumulus cell gap junctional communication (GJC) assay

The oocyte-cumulus cell GJC was assayed using fluorescent dye, calcein-AM [51]. Calcein-AM is nonfluorescent, electrically neutral, and highly lipophilic because of the acetoxymethyl groups in the molecule and can rapidly permeate into the cytoplasm through the cell membrane. Once inside the cell, nonspecific endogenous esterases cleave the lipophilic acetoxymethyl groups, producing calcein—a fluorescent, negatively charged molecule that is unable to leak out of cells across the plasma membrane, but is able to pass between cells connected via gap junctions. Cumulus-oocyte GJC was measured by quantitative fluorescence microscopy as the amount of calcein in the oocyte, transferred from the cumulus cells through gap junctions via passive diffusion.

COCs were cultured in HX medium or HX medium+FSH for 5, 10, 20, 30, 40, 50 or 60 min after which they were transferred to a solution of 1 µM calcein-AM freshly made up in a modified phenol red-free and protein-free HX Medium+polyvinylpyrrolidone (PVP, 0.3 mg/ml;), which is designated as modified medium, for 1 min, and were then transferred to calcein-AM free modified media and cultured for a further 3 min to allow for dye exchange between the cumulus cells and the oocyte. Unincorporated dye was then removed by three washes in calcein-AM-free modified medium. Prior to fluorescence analysis, COCs were completely denuded of their surrounding cumulus cells using vigorous pipetting. The intraoocyte fluorescence emission of calcein in pulsed oocytes was measured using a fluorophotometric-inverted microscope (Leica, Wetzlar, Germany). DOs in the experimental field of view were analyzed singularly and independently from neighboring oocytes. Fluorescence readings of DO in each replicate experiment are represented as relative fluorescence intensity compared to that of t = 0 h (%).

### PDE3A enzymatic activity assay

COCs were cultured in HX medium or HX medium+FSH for 0, 2, 4, 6 or 8 h after which they were denuded. DOs were collected and homogenized in an isotonic buffer containing 10 mM sodium phosphate buffer pH 7.2, 50 mM NaF, 150 mM NaCl, 2 mM EDTA, 5 mM 2-mercaptoethanol, 30 mM sodium pyrophosphate, 3 mM benzamidine, 5 mg/ml leupeptin, 20 mg/ml pepstatin, 2 mM phenylmethylsulfonyl fluoride, 1 mM microcystin and 0.5% triton X-100. The homogenate was centrifuged for 30 min at 14,000 *g* to obtain a soluble fraction [52]. PDE3A activity was assayed using 1 mM cAMP as substrate according to the method of Thompson [53]. Samples were assayed at 34°C in a final volume of 200 µl; the solution consisting of 40 mM Tris-HCl pH 8.0, 10 mM MgCl_2_, 5 mM 2-mercaptoethanol, 1 mg/ml BSA, 1 mM cAMP and 15 nM [^3^H]cAMP (Perkin-Elmer Life Sciences, Boston, MA).

### Statistics

Each oocyte maturation experiment was conducted at least three times with at least 30 oocytes per group per experiment. Data are reported as the mean percentage of GVBD ± SEM. Maturation frequencies were subjected to arcsin transformation and analysed statistically by ANOVA followed by Duncan's multiple range test and the other data no-transformed were analysed by the same test (SigmaStat; Systat Software, Inc., Richmond, CA, USA). A *p* value<0.05 was considered significant.

## Supporting Information

Figure S1 **Quantification of immunoblots corresponding to the** **Figure 3** **.**
(TIF)

Figure S2 **Quantification of immunoblots corresponding to the** **Figure 4** **.**
(TIF)

Figure S3 **Quantification of immunoblots corresponding to the** **Figure 5** **.** The densitometric data of the target proteins were normalized according to GAPDH to evaluate the relative abundance of them. Columns with different superscripts are significantly different (*P*<0.05).
(TIF)
