# Supplementary figures and images for: FSH Modulates PKAI and GPR3 Activities in Mouse Oocyte of COC in a Gap Junctional Communication (GJC)-Dependent Manner to Initiate Meiotic Resumption
Source: PLoS One. 2012 Sep 13;7(9):e37835. doi: 10.1371/journal.pone.0037835 (PMC3441574; doi:10.1371/journal.pone.0037835)

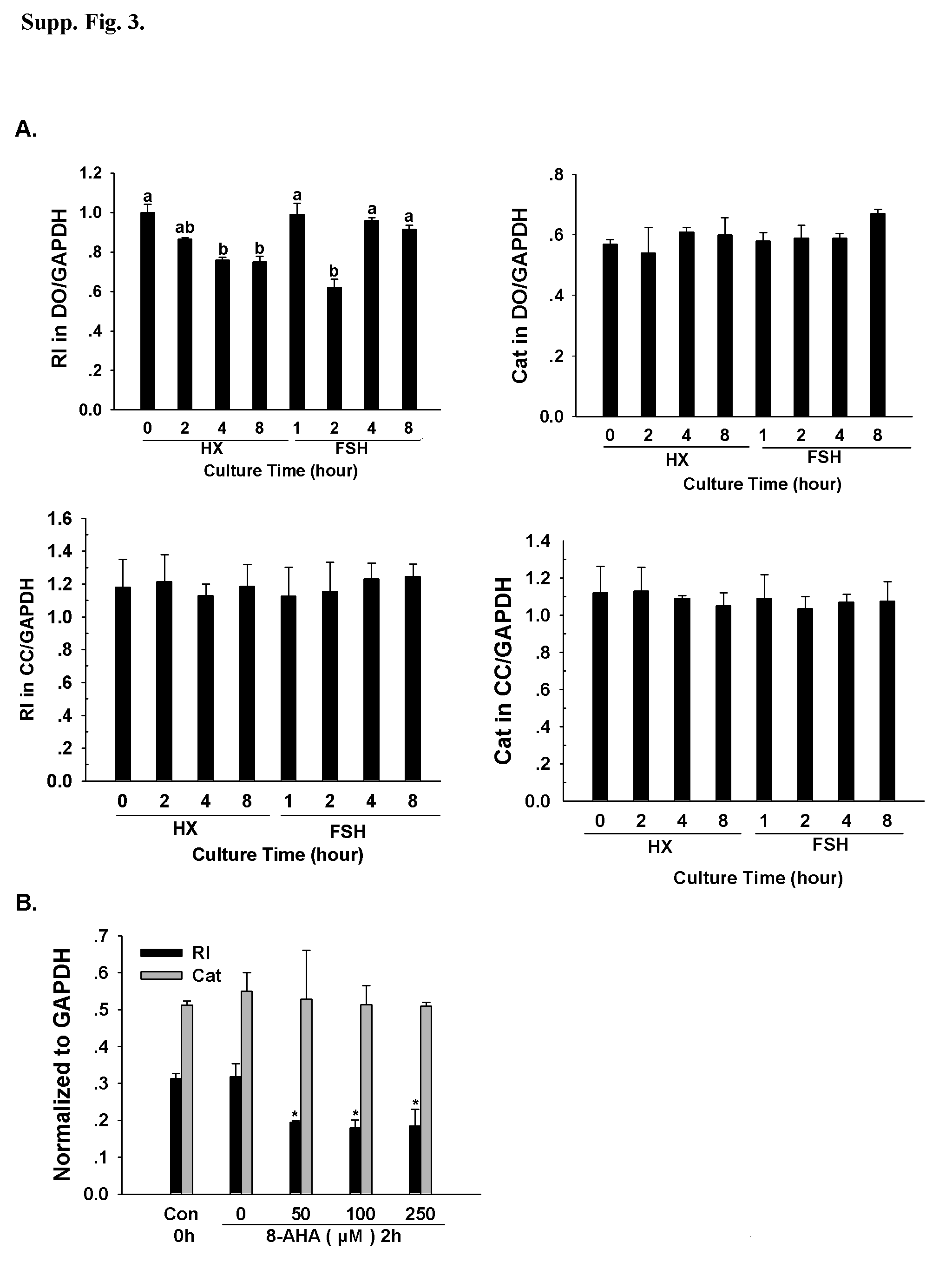

Supplement: Figure S1 — Quantification of immunoblots corresponding to the Figure 3 . (TIF) [file pone.0037835.s001.tif]
